# Characterizing Variability in Lung Cancer Outcomes and Influence of a Lung Diagnostic Assessment Program in Southeastern Ontario, Canada

**DOI:** 10.3390/curroncol30050368

**Published:** 2023-05-09

**Authors:** Shahad AlGhamdi, Weidong Kong, Michael Brundage, Elizabeth A. Eisenhauer, Christopher M. Parker, Geneviève C. Digby

**Affiliations:** 1 Department of Medicine, Division of Respirology, Queen’s University, Kingston, ON K7L 2V7, Canada; 2 Cancer Care and Epidemiology Research Unit, Queen’s University, Kingston, ON K7L 2V7, Canada; 3 Department of Oncology, Queen’s University, Kingston, ON K7L 2V7, Canada; 4 Department of Critical Care Medicine, Queen’s University, Kingston, ON K7L 2V7, Canada

**Keywords:** lung cancer, multidisciplinary clinic, system barriers, mortality, Ontario

## Abstract

Introduction: Regional variability in lung cancer (LC) outcomes exists across Canada, including in the province of Ontario. The Lung Diagnostic Assessment Program (LDAP) in southeastern (SE) Ontario is a rapid-assessment clinic that expedites the management of patients with suspected LC. We evaluated the association of LDAP management with LC outcomes, including survival, and characterized the variability in LC outcomes across SE Ontario. Methods: We conducted a population-based retrospective cohort study by identifying patients with newly diagnosed LC through the Ontario Cancer Registry (January 2017–December 2019) and linked to the LDAP database to identify LDAP-managed patients. Descriptive data were collected. Using a Cox model approach, we compared 2-year survival for patients managed through LDAP vs. non-LDAP. Results: We identified 1832 patients, 1742 of whom met the inclusion criteria (47% LDAP-managed and 53% non-LDAP). LDAP management was associated with a lower probability of dying at 2 years (HR 0.76 vs. non-LDAP, *p* < 0.0001). Increasing distance from the LDAP was associated with a lower likelihood of LDAP management (OR 0.78 for every 20 km increase, *p* < 0.0001). LDAP-managed patients were more likely to receive specialist assessment and undergo treatments. Conclusions: In SE Ontario, initial diagnostic care provided via LDAP was independently associated with improved survival in patients with LC.

## 1. Introduction

Lung cancer (LC) is the leading cause of cancer-related mortality worldwide [1]. In 2022, it is estimated that 30,000 Canadians will be diagnosed with LC and over 20,000 will die from it [2]. The high mortality associated with this malignancy is due to the aggressive nature of the disease and frequent late-stage disease at presentation [3], while diagnostic and treatment delays are also thought to be contributing factors [4].

In Canada, significant variability in LC outcomes exists [5]. Even within the province of Ontario, LC outcomes vary across health regions, formerly known as Local Health Integration Networks (LHINs), with 5-year LC survival ranging from to 22% to 32% in 2014–2018 [5], with the lowest survival noted in southeastern (SE) Ontario. Socioeconomic and geographic factors have been shown to contribute to the variability in LC survival. Populations experiencing worse outcomes include lower-income persons, recent immigrants, and rural residents, as well as First Nations, Inuit and Métis [6,7]. These potential disparities are of concern in the SE region, which serves a predominantly rural patient population with a higher representation of low-income households. Additionally, southeastern Ontario has the second-highest smoking rate, the highest proportion of seniors, and the fourth-largest Aboriginal population in the province [8].

In 2009, Lung Diagnostic Assessment Programs (LDAPs) were established in Ontario. Designed as rapid-assessment clinics, LDAPs help provide efficient and appropriate diagnostic and staging workup for patients with suspected LC [9,10]. LDAPs often also include patient navigation, psychosocial support, and a multidisciplinary team approach involving different specialists [11], which are reasons attributed to the finding that rapid-assessment models reduce patient anxiety and improve quality of life [12,13]. Habbous et al. studied the effects of LDAPs in LC diagnosis and treatment in Ontario from 2014 to 2016 and found that LDAPs are associated with improved time to diagnosis, specialist assessment, and treatment, with a trend towards improved survival but uncertainty regarding the contributing factors [10]. As such, the association between LDAP management and longer survival has not been demonstrated and needs to be further explored [14].

In 2016 when the SE-LHIN was identified as having the lowest 5-year LC survival in the province of Ontario, the SE-LDAP implemented several quality improvement efforts to provide more efficient, timely, and cost-effective care with a goal of improving patient care and outcomes [9,15,16,17]. In this study, we evaluate the association of LDAP management with LC outcomes, including survival in the SE region. Further, we characterize the variability in LC outcomes across SE Ontario in order to identify opportunities for improvement that specifically target identified barriers to care access.

## 2. Materials and Methods

### 2.1. Local Context

As of 2015, the population of the SE-LHIN was approximately 500,000 people, or 3.6% of the Ontario population. Every year, approximately 500 patients are diagnosed with LC in this region, with approximately 50% of patients seen through the LDAP, which consists of two clinical streams by which patients are assessed: a respirology LDAP stream through which 80–85% of patients are assessed and a parallel thoracic surgery stream, to which patients suspected of having resectable and operable lung cancer were triaged (15–20%).

### 2.2. Study Design

We conducted a population-based retrospective cohort study from 1 January 2017 to 31 December 2019. We identified patients with newly diagnosed LC through the Ontario Cancer Registry (OCR). We then linked this data to the SE-LHIN LDAP database to identify patients that received care through the LDAP.

### 2.3. Study Participants

Cases were defined as patients over 18 years of age with a new diagnosis of LC between 1 January 2017 and 31 December 2019, identified by first topography and morphology coding consistent with LC in OCR. Patients were excluded from the study if they did not have data in OCR, were diagnosed with LC recurrence (i.e., defined by clinical assessment, radiologically, or pathologically as recurrence rather than a new primary), were diagnosed outside of the study period, had a non-LC histology type, or resided outside of the SE-LHIN. One patient with synchronous diagnoses of adenocarcinoma and squamous cell carcinomas was excluded from the histologic-type summative data (Table 1) as well as the univariate and multivariate analyses (Table 2 and Table 3) and factors associated with LDAP management analysis (Table 4) where histologic data are used in the analysis.

### 2.4. Study Variables and Data Collection

#### 2.4.1. Independent Variables

Descriptive data were collected, including patient characteristics such as age, which was stratified into age groups (18–60, 61–70, 71–80, and >80 years), with the 18–60 age group used as reference; sex, which was stratified into male and female, with male used as reference group; geographic distance from the LDAP, which was stratified by distance (<50 km, 50–100 km, and >100 km), with <50 km used as reference group; and income quintile stratified into five groups 1 (lowest)–5 (highest) with lowest income quintile used as reference group. Income quintile was determined based on the patient’s postal code and adjusted household income data from Statistics Canada’s (StatCan) census data. Income quintile represents median adjusted household income by family size [18].

Data regarding disease characteristics included histologic subtype, which was stratified into adenocarcinoma, squamous cell carcinoma, poorly differentiated carcinoma, small cell carcinoma, large cell carcinoma, neuroendocrine not otherwise specified (NOS) and other, with adenocarcinoma subtype representing the reference group, and stage at diagnosis, which was stratified as stage I, II, III, IV and unknown with stage IV considered as the reference group. Other data collected were the number of imaging studies, biopsies, and hospital admissions during the diagnostic phase of LC, defined as the time from first abnormal imaging suspicious for LC to first LC treatment. Other system variables collected included first treatment received and specialist assessments during the diagnostic phase of LC. Details regarding procedures, hospital admission, investigations, and health-care encounters were extracted from billing codes in the OHIP system, complemented by data from the National Ambulatory Care Reporting System (NACRS) and the Canadian Institution for Health Information Discharge Abstract Database (CIHI DAD).

#### 2.4.2. Dependent Variables

Date of patient death within 2 years of diagnosis was obtained from the OCR. Overall median survival and 2-year overall survival were defined as the time from diagnosis to death at 2 years.

### 2.5. Definitions

LC diagnosis was obtained either through coding consistent with LC in the OCR or from date of clinical and radiographic assessment where no pathologic diagnosis was available. LC stage was defined as per TNM 8th edition classification [19], with stage 0 representing Tis (carcinoma in situ) N0M0.

The diagnostic phase was defined as the interval from first abnormal imaging to first treatment. The first treatment modality was defined as the initial cancer treatment received by patients and included any of: surgical resection; systemic therapy, which included targeted therapy, cytotoxic chemotherapy, or immunotherapy; radiation therapy, which included initial radiation to the chest and/or site of metastases; and chemoradiation, defined as concurrent combined modality as the initial treatment.

### 2.6. Study Outcome

To evaluate the association of LDAP management with LC outcomes in SE Ontario, we assessed for differences in 2-year overall survival between LDAP-managed and non-LDAP-managed cohorts.

To characterize the variability in LC outcomes across SE Ontario, we assessed for differences in patient, disease, and system characteristics between the LDAP-managed and non-LDAP-managed cohorts.

### 2.7. Statistical Approach

We used a Cox model to complete a time-to-event analysis whereby we evaluated the effect of LDAP-management on overall survival from time of diagnosis, after adjusting for other patient and disease characteristics associated with LC survival. Logistic regression was performed to assess the factors associated with LDAP management. Backward elimination analysis, with LDAP retained, was used to select explanatory factors and eliminate insignificant variables in logistic model and Cox model analyses.

Hazard ratio (HR) and odds ratio (OR) were used to describe the effect of factors in model-based analyses on mortality (Table 2 and Table 3) and LDAP management (Table 4), respectively. A value of HR or OR > 1 indicates increased hazard of mortality or odds of LDAP management for a specific level of the factor compared to the reference level, with specified magnitude; a value of HR or OR < 1 indicates an effect of opposite direction. A threshold of 0.05 was chosen to determine statistical significance. A *p*-value < 0.05 was deemed as statistically significant.

We considered the potential impact of lead-time bias affecting the results of the study on the relationship between LDAP management and favorable outcome for LC. We evaluated this possibility by analyzing survival from the date of first abnormal imaging (rather than from date of diagnosis). SAS for Windows 9.4 TS1M6 (Cary, NC, USA) was used to conduct statistical analyses.

The study was approved by the Queen’s University Health Sciences Research Ethics Board.

## 3. Results

**Figure 1:**
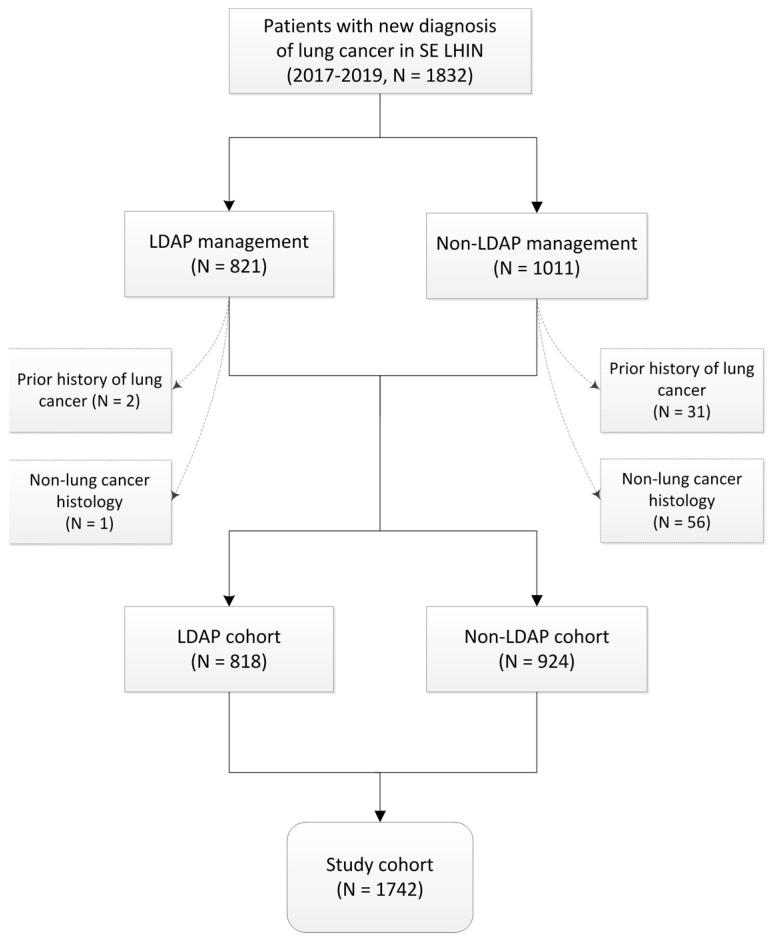
Flowchart of patient inclusion and exclusion criteria. Legend: LDAP, Lung Diagnostic Assessment Program; N, number of patients; SE LHIN, Southeastern Local Health Integration Network.

A total of 1832 patients were identified from the OCR database, of which 1742 met the inclusion criteria. Of these, 818 (47%) were managed through the LDAP, while 924 (53%) were from the non-LDAP cohort (Figure 1).

### 3.1. Patient and Disease Characteristics

Patient characteristics at the time of LC diagnosis for both LDAP and non-LDAP, cohorts are summarized in Table 1. Median age was similar in both cohorts at 71 years and the proportion of patients of female sex was also similar (54.2% and 51.7% in LDAP and non-LDAP cohorts, respectively). A greater proportion of patients in the LDAP cohort resided within 50 km of the LDAP (46.5% vs. 29.5%). Most patients in both cohorts were from income quintiles 2 and 3 (61.1% of all patients).

The majority of LC histology was adenocarcinoma (38.8% overall) followed by poorly differentiated carcinoma (29.8%), with similar distribution between LDAP and non-LDAP cohorts. The proportion of patients with adenocarcinoma increased over time from 2017 to 2019 (33.2% in 2017, 41.0% in 2018, 41.8% in 2019). There were more patients with advanced-stage LC in the non-LDAP cohort than the LDAP cohort (43.8% and 32.3%, respectively) (Table 1).

**Table 1:** Patient characteristics in LDAP and non-LDAP cohorts.

Characteristic	LDAP Cohort (N = 818)	Non-LDAP Cohort (N = 924)	Overall (N = 1742)
**Age**			
18–60	118 (14.4%)	161 (17.4%)	279 (16.0%)
61–70	288 (35.2%)	275 (29.8%)	563 (32.3%)
71–80	290 (35.5%)	334 (36.1%)	624 (35.8%)
>80	122 (14.9%)	154 (16.7%)	276 (15.9%)
median (IQR)	71 (64, 77)	71 (63, 78)	71 (64, 78)
**Sex** (female N/%)	443 (54.2%)	478 (51.7%)	921 (52.9%)
**Distance from LDAP (km)**			
<50	380 (46.5%)	272 (29.5%)	652 (37.4%)
50–100	405 (49.5%)	505 (54.7%)	910 (52.2%)
>100	33 (4.0%)	147 (15.9%)	180 (10.3%)
median (IQR)	56.9 (10.0, 76.6)	74.3 (36.8, 88.7)	70.8 (23.6, 84.4)
**Income Quintile**			
1 (lowest)	139 (17.0%)	185 (20.0%)	324 (18.6%)
2	238 (29.1%)	284 (30.7%)	522 (30.0%)
3	270 (33.0%)	272 (29.4%)	542 (31.1%)
4	130 (15.9%)	158 (17.1%)	288 (16.5%)
5 (highest)	41 (5.0%)	25 (2.7%)	66 (3.8%)
**Histology Type**			
Adenocarcinoma	346 (42.4%)	329 (35.6%)	675 (38.8%)
Squamous cell carcinoma	162 (19.8%)	134 (14.5%)	296 (17.0%)
Poorly differentiated carcinoma	195 (23.9%)	322 (34.8%)	517 (29.7%)
Small cell carcinoma	77 (9.4%)	94 (10.2%)	171 (9.8%)
Large cell carcinoma	5 (0.6%)	14 (1.5%)	19 (1.1%)
Neuroendocrine NOS	4 (0.5%)	9 (1.0%)	13 (0.7%)
Other	28 (3.4%)	22 (2.4%)	50 (2.9%)
**Stage (OCR best stage)**			
0/I	238 (29.1%)	144 (15.6%)	382 (21.9%)
II	68 (8.3%)	38 (4.1%)	106 (6.1%)
III	153 (18.7%)	114 (12.4%)	267 (15.3%)
IV	264 (32.3%)	404 (43.8%)	668 (38.4%)
Unknown	95 (11.6%)	224 (24.3%)	319 (18.3%)

Legend: IQR, interquartile range; LDAP, Lung Diagnostic Assessment Program; N, number of patients; NOS, not otherwise specified; OCR, Ontario Cancer Registry. Note: One patient with synchronous diagnoses of adenocarcinoma and squamous cell carcinomas was excluded from the histologic-type summative data.

### 3.2. Factors Influencing Lung Cancer Survival

Factors associated with probability of death within 2 years are presented in Table 2. In the unadjusted analysis, younger age (18–60), female sex and earlier LC stage were associated with a reduced risk of death. Factors associated with a higher probability of dying within 2 years were lower income quintile and non-adenocarcinoma histology. In the adjusted analysis, these variables remained significant.

Increased distance from patient residence relative to the LDAP was associated with an increased risk of death in the unadjusted analysis (HR 1.22 for 50–100 km, HR 1.28 for >100 km vs. reference < 50 km, *p* = 0.0045), but was not significant when LDAP management was forced into the adjusted analysis (*p* = 0.3302) (Table 2).

Comparing the LDAP and non-LDAP cohorts, 2-year overall survival was higher in the LDAP cohort (44.7%) than the non-LDAP cohort (29.8%). LDAP management was associated with a lower probability of dying at 2 years, which remained significant in the adjusted analysis (HR 0.76 for LDAP management compared with non-LDAP management, *p* < 0.0001) (Table 2).

**Table 2:** Factors associated with overall median and 2-year survival—unadjusted analysis and adjusted analysis from diagnosis.

Factor	N	Overall Survival		Unadjusted		Adjusted	
		Median (Months)	2-Year	HR (95% CI)	*p*	HR (95% CI)	*p*
**Age**							
18–60	279	15.8	41.9%	reference	**0.0062**	reference	**<0.0001**
61–70	563	12.7	37.9%	1.12 (0.93, 1.35)		1.26 (1.05, 1.53)	
71–80	623	11.1	35.2%	1.24 (1.03, 1.49)		1.60 (1.33, 1.93)	
>80	276	8.9	32.6%	1.41 (1.14, 1.74)		1.78 (1.43, 2.21)	
**Sex**							
Female	920	15.8	43.0%	0.68 (0.61, 0.77)	**<0.0001**	0.79 (0.70, 0.89)	**0.0001**
Male	821	8.6	29.7%	reference		reference	
**Distance from LDAP (kilometers)**							
<50	652	15	41.8%	reference	**0.0045**	reference	0.3302
50–100	909	10.2	34.7%	1.22 (1.07, 1.39)		1.10 (0.95, 1.26)	
>100	180	10.1	28.9%	1.28 (1.04, 1.56)		0.99 (0.80, 1.23)	
**Income Quintile**							
1 (lowest)	324	8.4	31.1%	2.27 (1.53, 3.36)	**0.0004**	2.01 (1.35, 3.01)	**0.0026**
2	522	12.4	38.2%	1.79 (1.21, 2.62)		1.56 (1.05, 2.33)	
3	541	11.2	34.8%	1.89 (1.28, 2.77)		1.75 (1.17, 2.61)	
4	288	11.7	39.4%	1.81 (1.22, 2.70)		1.69 (1.13, 2.53)	
5 (highest)	66	33.2	56.1%	reference		reference	
**Histology Type (OCR histology)**							
Adenocarcinoma	675	29.3	53.1%	reference	**<0.0001**	reference	**<0.0001**
Squamous cell carcinoma	296	14.3	37.1%	1.45 (1.21, 1.73)		1.49 (1.24, 1.80)	
Poorly differentiated carcinoma	517	4.1	22.3%	2.55 (2.21, 2.96)		2.13 (1.82, 2.48)	
Small cell carcinoma	171	6.7	12.0%	2.66 (2.18, 3.24)		1.75 (1.43, 2.14)	
Other	82	16.5	42.9%	1.27 (0.93, 1.73)		1.19 (0.87, 1.62)	
**Stage (OCR best stage)**							
0/I	381	-	75.8%	0.16 (0.13, 0.20)	**<0.0001**	0.16 (0.13, 0.20)	**<0.0001**
II	106	-	62.1%	0.24 (0.17, 0.32)		0.27 (0.19, 0.37)	
III	267	14	31.8%	0.50 (0.42, 0.59)		0.51 (0.43, 0.61)	
IV	668	4.4	14.9%	reference		reference	
unknown	319	6.0	30.6%	0.71 (0.61, 0.83)		0.63 (0.53, 0.74)	
**LDAP**							
No	924	7.5	29.8%	reference	**<0.0001**	reference	**<0.0001**
Yes	817	18.5	44.7%	0.61 (0.54, 0.69)		0.76 (0.67, 0.87)	

Legend: CI, confidence Interval: HR, hazard ratio; LDAP, Lung Diagnostic Assessment Program; N, number of patients; OCR, Ontario Cancer Registry. Note: One patient with synchronous diagnoses of adenocarcinoma and squamous cell carcinomas was excluded from the analysis.

Overall median survival (months) showed consistent findings, with higher survival in younger patients (age < 60), female sex, higher income quintile, adenocarcinoma subtype and LDAP management.

To evaluate the possibility of lead-time bias affecting the results, we also analyzed survival from the date of first abnormal imaging (rather than from date of diagnosis) and found that the data still demonstrated that LDAP management had a positive influence on survival (HR 0.72 for LDAP management compared with non-LDAP management, *p =* 0.0161) (Table 3).

**Table 3:** Factors associated with overall 2-year survival—unadjusted analysis and adjusted analysis from first abnormal imaging.

Factor	N	Overall Survival		Unadjusted		Adjusted	
		Median (Months)	2-Year	HR (95% CI)	*p*	HR (95% CI)	*p*
**Age**							
18–60	271	17.5	42.9%	reference	**0.0247**	reference	**<0.0001**
61–70	539	14.5	38.6%	1.13 (0.93, 1.36)		1.33 (1.09, 1.61)	
71–80	593	13	38.2%	1.21 (1.00, 1.45)		1.64 (1.36, 1.99)	
>80	258	11.3	34.2%	1.38 (1.11, 1.71)		1.80 (1.44, 2.25)	
**Sex**							
Female	875	19.1	45.3%	0.67 (0.60, 0.76)	**<0.0001**	0.78 (0.69, 0.89)	**0.0001**
Male	786	10.3	30.9%	reference		reference	
**Distance from LDAP (kilometers)**							
<50	630	17.1	43.5%	reference	**0.0074**	reference	0.6509
50–100	864	12.4	36.6%	1.20 (1.05, 1.37)		1.03 (0.90, 1.19)	
>100	167	12.1	29.9%	1.30 (1.05, 1.60)		0.94 (0.75, 1.17)	
**Income Quintile**							
1 (lowest)	302	10	33.3%	2.18 (1.46, 3.26)	**0.0019**	2.02 (1.34, 3.04)	**0.0048**
2	499	14.5	38.9%	1.78 (1.20, 2.64)		1.63 (1.08, 2.44)	
3	519	13	36.6%	1.88 (1.27, 2.78)		1.84 (1.22, 2.76)	
4	277	14.5	42.0%	1.74 (1.16, 2.62)		1.64 (1.09, 2.48)	
5 (highest)	64	35.4	59.4%	reference		reference	
**Histology Type (OCR histology)**							
Adenocarcinoma	648	31.4	54.5%	reference	**<0.0001**	reference	**<0.0001**
Squamous cell carcinoma	291	16.4	39.1%	1.43 (1.19, 1.71)		1.45 (1.20, 1.76)	
Poorly differentiated carcinoma	483	5.9	24.1%	2.48 (2.13, 2.88)		2.10 (1.79, 2.46)	
Small cell carcinoma	162	7.5	12.1%	2.82 (2.30, 3.44)		1.78 (1.45, 2.19)	
Other	77	22.6	46.5%	1.21 (0.87, 1.67)		1.13 (0.82, 1.57)	
**Stage (OCR best stage)**							
0/I	374	-	77.2%	0.15 (0.12, 0.18)	**<0.0001**	0.15 (0.12, 0.19)	**<0.0001**
II	105	-	63.8%	0.22 (0.16, 0.30)		0.25 (0.18, 0.35)	
III	261	14.9	32.1%	0.49 (0.41, 0.58)		0.51 (0.43, 0.61)	
IV	627	5.4	15.7%	reference		reference	
unknown	294	8	33.7%	0.63 (0.53, 0.75)		0.56 (0.47, 0.67)	
**LDAP**							
No	844	9.2	31.4%	reference	**<0.0001**	reference	**0.0161**
Yes	817	20.6	45.9%	0.61 (0.54, 0.69)		0.72 (0.63, 0.82)	

Legend: CI, confidence Interval: HR, hazard ratio; LDAP, Lung Diagnostic Assessment Program; N, number of patients; OCR, Ontario Cancer Registry. Note: 80 patients with no imaging study identified in OCR were excluded and one patient with synchronous diagnoses of adenocarcinoma and squamous cell carcinomas was excluded.

### 3.3. Factors Associated with LDAP Management

Patient factors associated with LDAP management are presented in Table 4. Patients aged 61–70 were more likely to be LDAP-managed than younger patients (OR 1.56, *p* = 0.0122), as were patients that were not in the lowest income quintile (*p* = 0.0209). LDAP management was associated with a higher likelihood of being diagnosed with early LC stage (OR > 2.18 for stage 0-III LC vs. reference stage IV, *p* < 0.0001), whereas unknown stage was associated with a lower likelihood of LDAP management (OR 0.73, *p* < 0.0001). Increasing distance from the LDAP was associated with a lower likelihood of LDAP management, with an OR of 0.78 for every 20 km increase, *p* < 0.0001.

**Table 4:** Factors associated with LDAP management.

Factor	N (%) with LDAP	OR (95% CI)	*p*-Value
**Age**			
18–60	118 (42.3%)	reference	**0.0122**
61–70	288 (51.2%)	1.56 (1.15, 2.13)	
71–80	289 (46.4%)	1.15 (0.85, 1.56)	
>80	122 (44.2%)	1.09 (0.75, 1.57)	
**Sex**			
Male	375 (45.7%)	reference	0.6808
Female	442 (48.0%)	0.96 (0.78, 1.18)	
**Distance from LDAP (kilometers)**			
<50	380 (58.3%)	reference	**<0.0001**
50–100	404 (44.4%)	0.55 (0.44, 0.69)	
>100	33 (18.3%)	0.14 (0.09, 0.22)	
**Income Quintile**			
1 (lowest)	139 (42.9%)	reference	**0.0209**
2	238 (45.6%)	1.24 (0.92, 1.67)	
3	269 (49.7%)	1.53 (1.13, 2.06)	
4	130 (45.1%)	0.97 (0.69, 1.37)	
5 (highest)	41 (62.1%)	1.35 (0.76, 2.44)	
**Stage**			
0/I	237 (62.2%)	2.41 (1.84, 3.17)	**<0.0001**
II	68 (64.2%)	2.76 (1.78, 4.34)	
III	153 (57.3%)	2.19 (1.63, 2.97)	
IV	264 (39.5%)	reference	
unknown	95 (29.8%)	0.73 (0.54, 0.98)	
**Histology Type (OCR histology)**			
Adenocarcinoma	346 (51.3%)	reference	0.0629
Non-adenocarcinoma	471 (44.2%)	0.82 (0.66, 1.01)	

Legend: CI, confidence interval; LDAP, Lung Diagnostic Assessment Program; N, number of patients; OCR, Ontario Cancer Registry; OR, odds ratio. Note: One patient with synchronous diagnoses of adenocarcinoma and squamous cell carcinomas was excluded from the analysis.

### 3.4. Influence of LDAP Management on Subsequent LC Care (Specialist Assessment, Treatment, and Health Resource Utilization)

#### 3.4.1. Specialist Assessment

**Figure 2:**
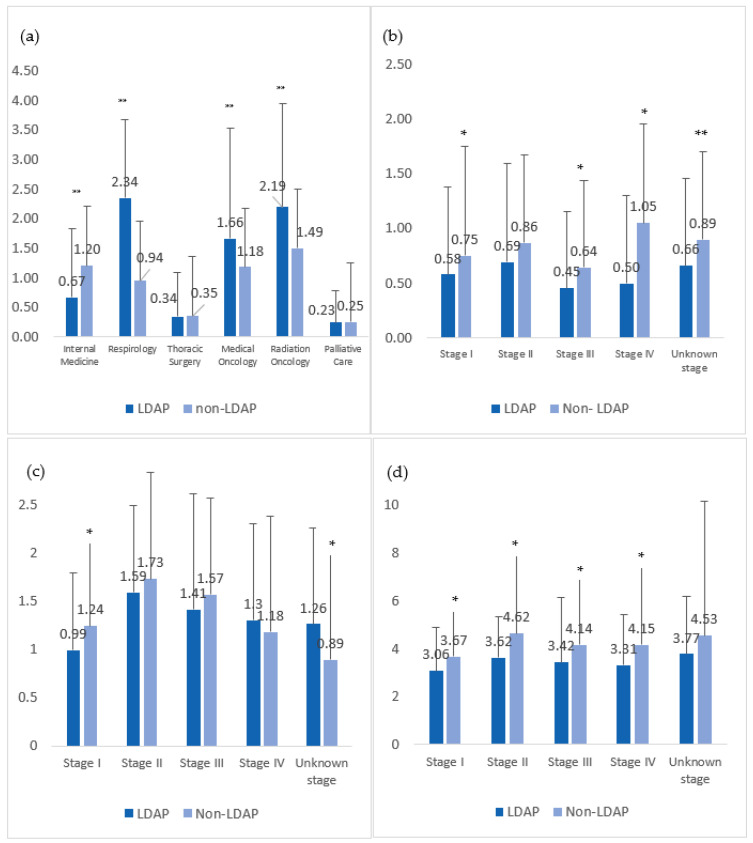
Influence of LDAP management on lung cancer care in diagnostic phase. (**a**) Average number of specialist assessments per patient ± SD. (**b**) Average number of hospital admissions ± SD. (**c**) Average number of biopsies per patient ± SD. (**d**) Average number of imaging studies per patient ± SD. Legend: LDAP, Lung Diagnostic Assessment Program; * = *p* < 0.05; ** = *p* < 0.001.

LDAP-managed patients had fewer visits to internal medicine specialists compared with the non-LDAP cohort (0.67 ± 1.15 visits/patient in the LDAP-managed cohort vs. 1.20 ± 1.68 visits/patient in the non-LDAP-managed cohort, *p* < 0.0001), but had more visits to respiratory specialists (2.34 ± 1.33 visits/patient LDAP vs. 0.94 ± 1.45 visits/patient non-LDAP, *p* < 0.0001) (Figure 2). Patients in the LDAP cohort also had significantly more specialist visits to both medical (MO) and radiation oncology (RO) than patients in the non-LDAP cohort (1.66 ± 1.86 visits with MO/patient, 2.19 ± 1.75 visits with RO/patient in the LDAP-managed cohort vs. 1.18 ± 1.61 visits with MO/patient, 1.49 ± 1.66 visits with RO/patient ±1.66 in non-LDAP cohort). Specialist visits to thoracic surgery and palliative care were similar between groups (Figure 2).

#### 3.4.2. Health Resource Utilization

The LDAP cohort had fewer hospital admissions per patient (0.55 ± 0.76 vs. 0.90 ± 0.89 admissions/patient in non-LDAP cohort, *p* < 0.0001) as well as fewer imaging studies per patient (3.34 ± 2.16 vs. 4.18 ± 3.79 in non-LDAP cohort, *p* < 0.0001). The average number of biopsies per patient was similar in both groups (1.25 ± 1.03 LDAP vs. 1.19 ± 1.14 non-LDAP, *p* = 0.33) (Figure 2).

#### 3.4.3. Treatment

Overall, patients with LC in the LDAP cohort had a higher probability of receiving treatment than those in the non-LDAP cohort (*p* < 0.0001). Furthermore, when analyzing each stage of LC, patients in stage I, stage IV and unknown-stage categories were more likely to receive treatment if managed through LDAP (*p* < 0.0001) (Table 5).

**Figure 3:**
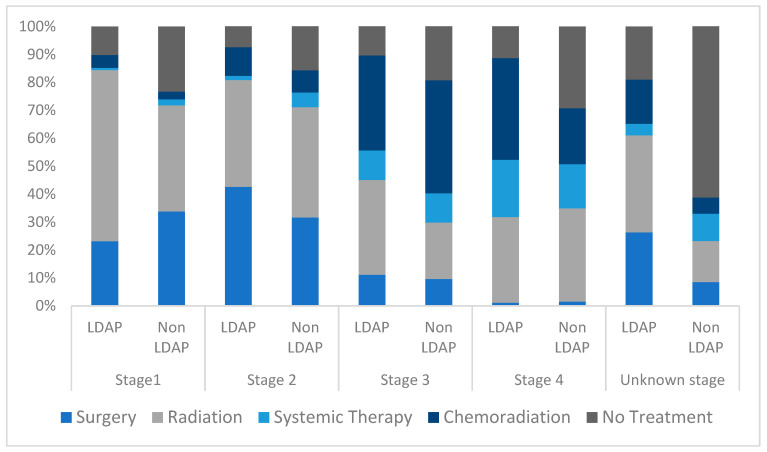
Percentage of first treatments received for lung cancer in LDAP and non-LDAP cohorts. Legend: LDAP, Lung Diagnostic Assessment Program.

The type of treatments received varied according to whether initial patient management occurred through the LDAP or not. Compared with the non-LDAP cohort, a greater proportion of stage I LC patients in the LDAP cohort received radiation therapy and a greater proportion of non-LDAP patients underwent surgery. Meanwhile, in stage II disease, a greater proportion of LDAP patients underwent surgery. In stage III disease, a greater proportion of LDAP patients received upfront radiation therapy alone, while slightly more patients underwent combined modality treatment in the non-LDAP cohort. A greater proportion of patients with stage IV LC received combined modality (chemoradiation) in the LDAP cohort whereas a greater proportion of patients managed outside of the LDAP received systemic therapy alone. In patients with unknown-stage LC, a higher percentage of patients in the LDAP cohort underwent surgery, radiation, and combined modality treatment (Table 5 and Figure 3).

## 4. Discussion

Many patient and disease factors are likely to influence LC survival. We found that while various patient and disease characteristics influence 2-year overall LC survival in SE Ontario, management of patients suspected of having LC through a specialized rapid-access clinic (the LDAP) is an independent factor influencing survival. We also found that increasing distance from the LDAP had the greatest negative association with LDAP management; however, it was not a factor influencing survival in the adjusted analysis. These results suggest a complex interplay between health system characteristics and patient factors that collectively influence survival.

It is well documented that significant variability in LC outcomes exist worldwide [1] and across provinces in Canada [5]. Even within the province of Ontario, significant variability in LC outcomes exist across health regions, not attributable to any single patient, disease, or system factor [14]. Beyond patient and disease factors previously shown to be associated with survival outcomes, we found that there are system factors even within a single health region that further affect LC outcomes. For example, we found that increasing distance from the LDAP had the greatest negative association with LDAP management.

Rapid-assessment clinics such as the LDAP have been shown to have several potential benefits for the management of patients undergoing evaluation for suspected LC, in that they have been shown to reduce delays in diagnosis and treatment [9,12,15] and provide patient-centered care [13,16]. However, demonstrating improved survival benefit has been more elusive [10,12]. Habbous et al. examined the effect of LDAPs on the diagnosis and treatment of patients with LC in Ontario, Canada from 2014–2016 [10]. They similarly found that patients were more likely to be diagnosed in an LDAP if they lived closer to an LDAP [10]. While they also found that Ontario LDAPs may be associated with improved survival, this finding was thought to be confounded by the large numbers of patients with urgent presentations to hospitals [10]. Our study demonstrates the survival benefit associated with LDAP management in SE Ontario. Interestingly, while increasing distance from the LDAP was associated with a lower likelihood of LDAP management, the survival benefit associated with LDAP management remained significant in the adjusted model (HR 0.61 in unadjusted model vs. 0.76 in adjusted model, both significant). This suggests that LDAP management itself imparts a survival benefit unrelated to distance and highlights the complex interplay between proximity to the LDAP and LDAP management itself, of particular importance to rural health regions such as SE Ontario given the geographic dispersion and predominantly rural patient population [7,8].

Previous studies have highlighted the impact of social determinants of health on LC outcomes [6,14]. Patients of lower socioeconomic status are almost twice as likely to be diagnosed with LC and tend to have more advanced disease at diagnosis [6]. Even when diagnosed at an earlier stage, patients in lower-income groups are less likely to receive curative treatments, which ultimately impacts survival outcomes [6]. Our study suggests a complex relationship between income quintile and distance from the LDAP: lower income quintile and increased distance from the LDAP were associated with lower likelihood of LDAP management and higher probability of death; however, in the adjusted analysis with LDAP as a variable, distance from the LDAP was no longer significant while income remained significant, raising the possibility that higher income quintile may overcome the barrier of distance from the LDAP.

Given that distance from the LDAP alone does not account for the survival benefit associated with LDAP management, we explored other factors associated with LDAP management and survival. Habbous et al. demonstrated significant regional variability in factors associated with diagnosis in an LDAP [10]. Similar to Habbous et al., we found that patients with earlier-stage disease (I-III) were more likely to be managed in the LDAP. We also found that patients aged 61–70 and those not in the lowest income quintile to be more likely to receive LDAP management in SE Ontario. Furthermore, we found that adjusting for patient and disease factors alone did not account for the difference in survival associated with LDAP management, suggesting a direct impact of LDAP management on survival.

We considered the possibility of lead-time bias affecting the results of the study on the relationship between LDAP management and favorable outcome for LC. However, we evaluated this possibility by analyzing survival from the first abnormal imaging and found that the data still supported the conclusion that LDAP management had a positive influence on survival.

### 4.1. Association between LDAP Management and Treatment

One of the factors that may have led to improved survival in the LDAP cohort was that patients receiving LDAP management were also more likely to receive subsequent treatment, which has not previously been explored. For all stages of disease, patients in the LDAP cohort were more likely to receive treatment than patients in the non-LDAP cohort. Not surprisingly, and in keeping with previous work, we found that patients with any stage of untreated lung cancer have a higher mortality rate [20,21] than patients who undergo treatment.

We found that for each stage of disease, initial patient management through the LDAP also influenced the treatment that patients received subsequent to their diagnosis, even after accounting for stage. Patients with stage I LC in the LDAP cohort were more likely to receive radiation therapy than those in the non-LDAP-managed cohort. In the LDAP cohort, we note that patients were older and more likely to require concurrent management with respiratory specialists, likely suggestive of increased comorbid disease burden in this cohort reflective of more advanced pulmonary disease. Respiratory specialists have an important role in the management of patients with suspected LC, including facilitating initial diagnosis, assessment of patient fitness to undergo diagnostic and treatment interventions, and management of common complications of LC [22,23]. Optimization of comorbid lung disease such as chronic obstructive pulmonary disease (COPD) has been associated with improved perioperative outcomes in patients with early-stage LC [24,25,26]. In fact, one study showed that co-diagnosis of COPD and LC is associated with less curative treatment in early-stage disease, less palliative treatment in advanced-stage disease, and overall worse outcomes [27,28]. Given the benefits of respiratory specialist assessment in LC care, it is possible that the increased likelihood of respiratory specialist management in patients managed through the LDAP had an influential role in contributing to the observed improved survival.

In this study, approximately 15% of patients referred to the LDAP were managed by a thoracic surgery clinical pathway to which patients with suspected earlier-stage/resectable disease and fewer medical comorbidities are triaged. However, it is possible that even within this thoracic surgery pathway, patients had contraindications to proceeding with surgical resection, which may contribute to a higher proportion of LDAP-managed patients receiving radiation therapy for stage I disease.

In stage IV disease, patients were more likely to receive concurrent combined-modality therapy (i.e., with chemotherapy and radiation) if managed through LDAP, thought to be due to the multidisciplinary approach in our LDAP. Concurrent treatment may improve overall survival [29], although further studies are needed.

### 4.2. Identifying Barriers to LDAP Management

Given that LDAP management is associated with improved survival, identifying specific system barriers to LDAP management could help guide strategies to ensure that a greater proportion of patients receive the benefits associated with LDAP management. Considering that only about 50% of patients in southeastern Ontario are managed through LDAP, there is substantial room for improvement in LDAP access.

Distance to the LDAP was strongly associated with the likelihood of LDAP management. It is worth noting that geographic distance may underestimate the true distance traveled on roadways and would not account for other costs that may impact traveling patients. As such, strategies that seek to overcome this barrier would likely have a significant regional impact. These strategies could include implementing LDAP outreach clinics that bring the benefits of coordinated specialist LC care closer to patients in the community, as well as increasing the use of virtual care in LDAPs. In response to these data, we established an LDAP outreach clinic at a local community hospital in the region, which has allowed us to increase LDAP capacity and reduce distance traveled for patients to hopefully mitigate this barrier and improve access to care.

Habbous et al. showed that patients with advanced disease and urgent presentations are less likely to receive LDAP management [10]. We similarly found that LDAP-managed patients were more likely to have earlier-stage LC (OR > 2.18 for stages I–III vs. reference stage IV, *p* < 0.0001). Although histology type was not a statistically significant factor affecting LDAP management, there was a trend towards non-adenocarcinoma histologic subtype having a lower OR for LDAP management (non-adenocarcinoma OR 0.82 with *p* = 0.06). Given that patients with non-adenocarcinoma subtype tend to have more aggressive disease and higher mortality, this may be a signal for the role that other factors play in likelihood of referral and management through LDAP, such as smoking, rural dwelling, and stage of disease.

Interestingly, we also found that half of patients with stage I and II disease in the SE region were not managed through the LDAP. Of these, over a third underwent surgical resection, suggesting that these patients were managed by direct referral to thoracic surgery without formal enrollment in the LDAP. While we did not observe significant differences in 2-year LC survival between groups (75.5%, 65.2% in stage I and II LDAP cohort vs. 76.3%, 56.7% in stage I and II non-LDAP cohort, respectively), there are other benefits to LDAP management that include standardized care that is evidence-based, improved timeliness of care, and nurse navigation during diagnostic phase of LC, which has been associated with positive patient outcomes, including patient satisfaction and reduced anxiety [9,16,22,23].

### 4.3. Limitations

The observed data are consistent with some degree of selection bias in referring patients for LC management. Although the analysis considered known patient, disease, and system attributes, it is possible that differences in unmeasured or latent variables between the two cohorts could also account for some of the observed differences in outcome.

One of the other challenges with evaluating the differences in patient outcomes relative to LDAP management is the fact that the distinction between LDAP-managed and non-LDAP-managed patients is not always clear. For example, patients may be referred to the outpatient LDAP clinic, but may not be able to be assessed due to factors such as being an inpatient at the time of referral or patient deterioration requiring admission to hospital prior to or after LDAP consultation but prior to diagnosis. In this study, these patients were excluded from the LDAP cohort, and thus it is possible that the outcomes of these patients negatively influenced the non-LDAP cohort.

We note that some patients in our analysis had LC but did not have accurate staging available. Although this may be a data-quality issue within the OCR as our LDAP database often had more specific data for stage than was available through the OCR (LDAP database 2.8% unknown stage, OCR data 11.6% unknown stage), it is also possible that unknown stage may be an indicator of patient or disease factors that influence the ability of staging investigations to be done (e.g., unwillingness or inability to undergo staging investigations). As such, it is difficult to identify the factors affecting LDAP referral and overall survival for this cohort of patients.

We also attempted to evaluate the impact of LDAP on timeliness of care compared to the non-LDAP pathway. Studies have previously shown that LDAP management improves time to staging investigation, treatment, and specialist assessment [9,12,15]. However, we found that the OCR data that we used in this study were inconsistent in definitions of timeliness of care, which limited our analysis of this key system factor.

It is worth noting that the previous study by Habbous et al. examined the effects of DAPs from 2014 to 2016, while our updated analysis evaluates the period of 2017–2019, following several quality improvement initiatives within our LDAP. These have included implementing standardized radiologist reporting and specialist referral recommendations for patients with imaging suspicious for LC [30], implementing standardized triage pathways to expedite diagnostic and staging investigations [9], and the establishment of a multidisciplinary LC clinic to expedite oncology assessment [11,15]. Thus, direct comparison between our data and the data from that study is not possible.

Lastly, although we have demonstrated the benefits of LDAP management in SE Ontario, these findings may not be generalizable to other health regions. In fact, given that significant regional variability in LC outcomes exist across Ontario [14], a regional approach to identifying and addressing barriers to optimal LC outcomes is required.

## 5. Conclusions

Lung cancer remains a leading cause of death. However, outcomes may be improved through management via regional rapid-assessment programs such as the LDAP. In SE Ontario, initial care provided through an LDAP is an independent system factor affecting LC survival. Additionally, LDAP management appears to influence treatment, with more treatment options presented to patients who were managed via the LDAP than those that were not. Potential contributing factors for the improved survival outcomes seen in LDAP-managed patients include access to cancer treatments, receiving respirologist consultation, and undergoing guideline-appropriate staging. Improvement in initiatives that seek to overcome geographic barriers to LDAP management and address other system barriers to LDAP management may help improve access to regional LDAP care and further improve LC survival in the region.

## Figures and Tables

**Table 5:** First treatments received for lung cancer in LDAP and non-LDAP cohorts.

	LDAP		Non-LDAP	
	N (%)	95% CI	N (%)	95% CI
Stage I (*p* < 0.0001)				
Surgery	55 (23.1%)	16.9–30.8%	48 (33.8%)	24.5–44.5%
Radiation Therapy	146 (61.3%)	53.0–69.1%	54 (38.0%)	28.3–48.8%
Systemic Therapy	2 (0.8%)	0.2–4.2%	3 (2.1%)	0.5–8.0%
Chemoradiation	11 (4.6%)	2.2–9.5%	4 (2.8%)	0.8–9.0%
No Treatments	24 (10.1%)	6.1–16.2%	33 (23.2%)	15.4–33.4%
Stage II (*p* = 0.4160)				
Surgery	29 (42.6%)	28.5–58.1%	12 (31.6%)	16.2–52.4%
Radiation Therapy	26 (38.2%)	24.8–53.8%	15 (39.5%)	22.1–59.9%
Systemic Therapy	1 (1.5%)	0.2–11.4%	2 (5.3%)	1.0–22.8%
Chemoradiation	7 (10.3%)	4.1–23.5%	3 (7.9%)	2.0–26.3%
No Treatments	5 (7.4%)	2.5–19.8%	6 (15.8)	5.9–35.8%
Stage III (*p* = 0.0627)				
Surgery	17 (11.1%)	6.1–19.3%	11 (9.6%)	4.6–19.1%
Radiation Therapy	52 (34.0%)	25.0–44.3%	23 (20.2%)	12.3–31.4%
Systemic Therapy	16 (10.5%)	5.6–18.6%	12 (10.5%)	5.2–20.2%
Chemoradiation	52 (34.0%)	25.0–44.3%	46 (40.4%)	29.4–52.4%
No Treatments	16 (10.5%)	5.6–18.6%	22 (19.3%)	11.6–30.4%
Stage IV (*p* < 0.0001)				
Surgery	3 (1.1%)	0.3–4.4%	6 (1.5%)	0.5–4.0%
Radiation Therapy	81 (30.7%)	23.9–38.4%	135 (33.4%)	27.7–39.7%
Systemic Therapy	54 (20.5%)	14.8–27.5%	64 (15.8%)	11.7–21.1%
Chemoradiation	96 (36.4%)	29.2–44.2%	81 (20.0%)	15.4–25.6%
No Treatments	30 (11.4%)	7.3–17.4%	118 (29.2%)	23.8–35.3%
Unknown Stage (*p* < 0.0001)				
Surgery	25 (26.3%)	16.5–39.2%	19 (8.5%)	4.8–14.6%
Radiation Therapy	33 (34.7%)	23.5–47.9%	33 (14.7%)	9.7–21.8%
Systemic Therapy	4 (4.2%)	1.3–13.1%	22 (9.8%)	5.8–16.2%
Chemoradiation	15 (15.8%)	8.4–27.6%	13 (5.8%)	2.9–11.2%
No Treatments	18 (18.9%)	10.8–31.2%	137 (61.2%)	52.6–69.1%
Overall (*p* < 0.0001)				
Surgery	129 (15.8%)	12.8–19.3%	97 (10.5%)	8.1–13.3%
Radiation Therapy	338 (41.3%)	37.0–45.8%	260 (28.2%)	24.5–32.1%
Systemic Therapy	77 (9.4%)	7.1–12.4%	103 (11.2%)	8.8–14.1%
Chemoradiation	181 (22.1%)	18.6–26.1%	147 (15.9%)	13.1–19.3%
No Treatments	93 (11.4%)	8.8–14.5%	317 (34.3%)	30.4–38.5%

Legend: CI, confidence interval; LDAP, Lung Diagnostic Assessment Program; N, number of patients.

## Data Availability

The datasets generated during and/or analyzed during the current study are not publicly available, but are available from the corresponding author on reasonable request.
